# Advanced systemic amyloidosis secondary to metastatic renal cell carcinoma

**DOI:** 10.3332/ecancer.2020.1156

**Published:** 2020-12-15

**Authors:** Maria Asunción Algarra, Maria José Juan Fita, Sergio Sandiego, Héctor Augusto Aguilar, Pablo Álvarez, Mateo Quispe, Antonio Salvador, Adoración Egido, Javier Lavernia, Isidro Machado, José Rubio-Briones, Miguel Ángel Climent

**Affiliations:** 1 Servicio de Oncología Médica, Fundación Instituto Valenciano de Oncología (IVO), Calle Profesor Beltrán Báguena, 8, 46009, Valencia, Spain; 2 Servicio de Cardiología, Fundación Instituto Valenciano de Oncología (IVO), Calle Profesor Beltrán Báguena, 8, 46009, Valencia, Spain; 3 Servicio de Medicina Interna, Fundación Instituto Valenciano de Oncología (IVO), Calle Profesor Beltrán Báguena, 8, 46009, Valencia, Spain; 4 Servicio de Anatomía Patológica, Fundación Instituto Valenciano de Oncología (IVO), Calle Profesor Beltrán Báguena, 8, 46009, Valencia, Spain; 5 Servicio de Urología, Fundación Instituto Valenciano de Oncología (IVO), Calle Profesor Beltrán Báguena, 8, 46009, Valencia, Spain; a https://orcid.org/0000-0003-2105-8597

**Keywords:** nephrotic syndrome, renal cell carcinoma, AA amyloidosis, secondary amyloidosis, nephrectomy

## Abstract

Secondary amyloidosis is a rare complex complication related to chronic inflammatory disease. This complication is sparsely associated to malignant neoplasms. Renal cell carcinoma (RCC) is the most common solid organ malignancy related with this paraneoplastic syndrome. Some case reports have described stabilisation or even remission of amyloidosis with cytoreductive nephrectomy. Majority of those reports were based on locally advanced RCC. We report the first case of early aggressive systemic secondary amyloidosis in high-volume metastatic RCC. The subject was diagnosed with metastatic RCC within 6 months of secondary amyloidosis; on month 5 of initiation of targeted therapy (pazopanib) developed nephrotic syndrome with a heavy proteinuria (>18 g/day), severe hypoalbuminaemia (1.53 g/dL), intense and progressive oedema, severe pancolitis and mild dyspnoea with hypotension. A colon biopsy and the immunohistochemistry confirmed the histological diagnosis of a secondary amyloidosis. The multidisciplinary tumour board decided to perform cytoreductive nephrectomy in order to reduce the pro-inflammatory status. Pathology report showed a complete resection of clear cell RCC plus renal amyloid deposits. The patient died within 4 days of surgery due to multiorgan failure.

## Introduction

Secondary amyloidosis, also called AA amyloidosis, is a rare complex complication related to chronic inflammatory disease. This complication is sparsely associated to malignant neoplasms. Renal cell carcinoma (RCC) is the most common solid organ malignancy related with this paraneoplastic syndrome.

Untreated AA amyloidosis has a significant morbidity and mortality specifically due to end-stage renal disease. A successful treatment of the underlying inflammatory disease, including complete removal of the primary tumour has demonstrated stabilisation or even remission of amyloidosis.

We report the first patient with early aggressive systemic secondary amyloidosis in high-volume metastatic RCC. A literature review on paraneoplastic amyloidosis is provided.

## Case report

A healthy 48-year-old Caucasian man presented to Emergency Room with asthenia, anorexia and weight loss over last 6 months. Past Medical History was relevant for lactose intolerance. Family History showed a second degree relative with gastric cancer. A thoraco-abdominopelvic CT scan was performed with diagnosis of right renal mass (14 cm), renal vein thrombosis and synchronic pulmonary metastasis (largest 2 cm on right inferior lobe). A lung biopsy confirmed clear RCC metastasis.

At systemic treatment, initiation parameters show a poor risk prognosis metastatic RCC as per Heng criteria grade 3 anaemia: 6.4 g/dL (>12 g/dL), thrombocytosis: 641,000/μL (range 150,000–400,000/μL), normal lactate dehydrogenase (LDH): 244 U/L (range 140–280 U/L), normal Calcium: 8.8 mg/dL (range 8.5–10.2 mg/dL) and Karnofsky Index: 80% (range 80%–100%). Treatment with Pazopanib 800 mg/day was started in an outside hospital.

At first assessment, a stable disease was observed (stabilisation of the right renal mass but an increase of less than 20% by Response Evaluation Criteria In Solid Tumors (RECIST) 1.1 criteria of the target lung metastasis). Further follow-up showed a disease progression of several lung metastasis. A second-line therapy was ordered with immune checkpoint inhibitor (nivolumab). Before the patient could start nivolumab, he developed a decay on his performance status, grade 2 diarrhoea (up to seven stools per day) and whole-body oedema. A diagnosis of nephrotic syndrome with non-infectious diarrhoea was obtained (hypoalbuminaemia: 1.53 g/dL (range 3.4–5.4 g/dL), a severe 24-hour proteinuria: 7,610 mg/day (range 0–80 mg/day), with preserved renal function: estimated glomerular filtration rate (eGFR) > 90 ml/minute/1.73 m^2^ (range 90–130 ml/minute/1.73 m^2^), an alteration of haemostasis: Quick index 75% (range 80%–120%) and a negative stool culture).

**Figure 1.**
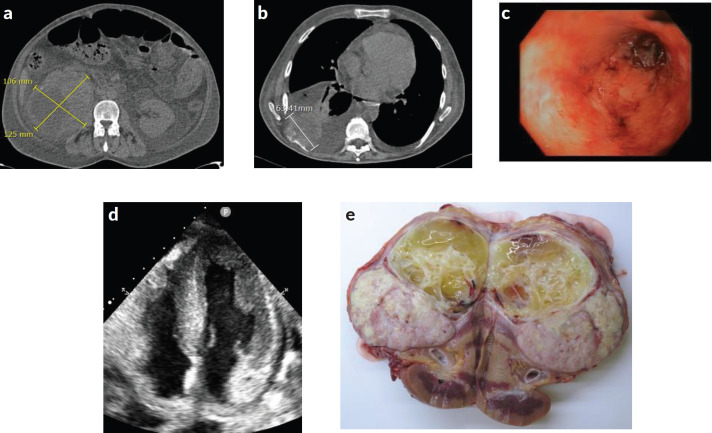
(a) and (b): Contrast-enhanced whole-body CT scan revealing stabilisation of the right renal mass, new bilateral pleural effusion and new pericardial effusion with an increase in the size of the lung metastasis (6 cm). (c): Lower gastrointestinal endoscopy demonstrating a severe pancolitis. (d): Echocardiography showing cardiac amyloidosis pattern. Increase in the thickness of the interventricular septum and walls of the ventricles. (e): Surgically removed piece from nephrectomy showing renal tumour.

Further work-up was performed with contrast-enhanced whole-body CT scan **(**Figure 1a–b**)** which revealed bilateral pleural effusion and pericardial effusion and increase in target lung metastasis size (6 cm). Also, lower gastrointestinal endoscopy was performed (Figure 1c). It detected a severe pancolitis. Autoimmune tests were negative (ANA, ANCA, IgA and IgG negative antiendomisio and proteinogram; erythrocyte sedimentation rate (ESR) 90).

**Figure 2.**
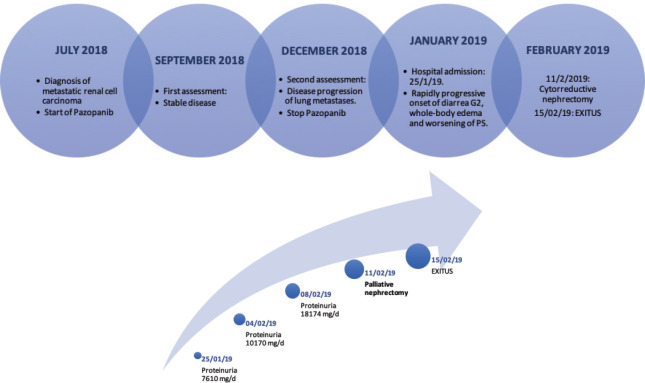
(a) and (b): First graph shows the timeline of the case. The second graph shows the progression of proteinuria over time.

At worsening of his condition (proteinuria (18,174 mg/day) and diarrhoea (Grade 3)) treatment with corticosteroids (1 mg/kg/day), albumin and furosemide was started while awaiting biopsy results. An improvement in diarrhoea (Grade 1) was observed, but the proteinuria worsened (Figure 2b).

**Figure 3.**
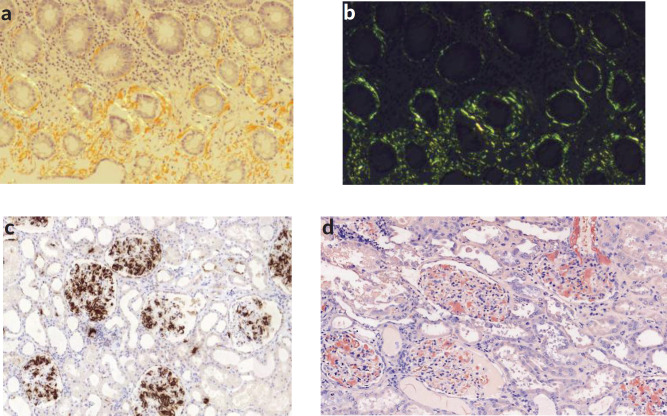
(a): Congo red stain of colon mucosa showing periglandular amyloid deposits. (b): Green birefringence of colon mucosa was observed by polarisation microscopy. (c): Immunohistochemistry study for amyloid AA showing strong and diffuse amyloid AA deposits in almost all the glomerulus and around blood vessels. (d): Congo red stain showed red deposition in almost all the glomerulus and around blood vessels.

The colon biopsy and the immunohistochemistry confirmed the histological diagnosis of a secondary amyloidosis (AA subtype): Congo red stain (Figure 3a) and apple green birefringence under polarised light were positive (Figure 3b).

Echocardiogram revealed advanced cardiac amyloidosis pattern, with an increase in the thickness of the interventricular septum, walls of the ventricles and right atrium and low cardiac output: left ventricular ejection fraction (LVEF) < 50% (range 50%–70%) (Figure 1d).

Our multidisciplinary tumour board decided to perform cytoreductive nephrectomy in order to reduce the pro-inflammatory status. A palliative right nephrectomy was performed without major intraoperative complications. During postoperative recovery, the patient developed haemodynamic shock, oligoanuria and acute kidney injury that did not respond to pharmacological treatment in the intensive care unit, precluding any additional treatment. The patient was deceased within 4 days of surgery due to multiorgan failure (Figure 2a).

RCC with sarcomatoid features was confirmed. The immunohistochemistry study for amyloid AA showed strong and diffuse amyloid AA deposits (in almost all the glomerulus, around blood vessels and the perinephritic fat) (Figures 1e, 3c and d).

## Discussion

AA amyloidosis is the worst potential complication of any chronic inflammatory condition. The most common underlying causes are chronic inflammatory diseases such as inflammatory arthritis, sarcoidosis, familial Mediterranean fever, chronic infections, inflammatory bowel disease such as Crohn’s disease and ulcerative colitis. However, malignant neoplasms are rarely reported to be associated with AA amyloidosis [1–4].

Malignant neoplasms are the underlying inflammatory driver in <7% of AA amyloidosis [3, 4]. One reason for the low incidence is the rapid progression of malignancies that causes the death of the majority of patients before significant systemic deposition of amyloid proteins in tissues. Large autopsy series suggests that RCC is clearly the most common solid organ malignancy to cause AA amyloidosis, representing 25%–42% of reported cases. But, conversely, amyloid deposits are only seen in 2.1%–3.2% of patients who died with a RCC [3, 4].

AA amyloidosis occurs in patients with high levels of serum amyloid A protein (SAA), which is produced by liver cells in response to signals from several pro-inflammatory cytokines such as IL-6, IL-1 and TNF-a. Renal cell tumours are known to produce high levels of tumour-derived IL-6 which can result in increased circulating SAA [5]. Systemic AA amyloidosis involves the deposit of amyloid protein in tissues. These amyloid proteins are derived from fragments of the SAA.

SAA is a dynamic acute-phase reactant protein and circulating levels can increase from a baseline of <3 mg/L to as high as 2,000 mg/L. During an inflammatory stimulus, high levels of SAA can remain persistently elevated. But, once the stimulus ends, there is a decline of circulating with a half-life of <24 hours [6, 7]. There are several possible mechanisms by which high levels of SAA in patients with malignant neoplasms can lead to amyloidosis. Malignant cells can directly produce SAA [8], or secrete pro-inflammatory cytokines [9] which signal the liver to produce SAA. Pro-inflammatory cytokines can also be produced by anti-tumour lymphocytes or macrophages. A combination of these three possibilities can be involved [10].

Maintained high circulating levels of SAA are a requisite for the development of AA amyloidosis but are clearly not sufficient. It is believed that there may be other factors involved such as extracellular matrix components, macrophage factors and SAA isotype. However, the role of these other factors is not entirely clear.

Common sites of involvement in secondary amyloidosis are the kidneys. Renal involvement most often presents as asymptomatic proteinuria or clinically apparent nephrotic syndrome. Other typical manifestations are hepatomegaly with or without splenomegaly, cardiac involvement that can lead to diastolic dysfunction, peripheral and autonomic neuropathy and impaired coagulation [11].

The definitive method for diagnosis of amyloidosis is tissue biopsy, although the presence of amyloidosis may be suggested by the history and clinical manifestations, even though it is not always a first option in differential diagnosis. Biopsies can be obtained from either clinically uninvolved sites, such as subcutaneous fat, minor salivary glands or rectal mucosa; or from dysfunctional organs. Congo red staining and examination using polarising microscopy has an overall sensitivity of 57% to 85% and a specificity of 92% to 100% for secondary amyloidosis. A review of 20 reported cases of the co-existence of amyloidosis and localised RCC has been published [12]. In two cases, the amyloidosis was of type AL (amyloid light-chain amyloidosis), but these patients had a concomitant myeloma and, in the other patients, the amyloid was of type AA. Amyloid deposits were found to occur in various tissues at extracellular sites: kidney, liver, spleen, adrenals and mucosa of the upper respiratory tract. Four cases of retroperitoneal amyloid deposits have also been reported.

We report the first English-language case of systemic AA amyloidosis due to metastatic RCC with high-volume metastatic disease. A single case report on RCC with muscle metastasis and secondary amyloidosis has been reported in Japanese language [14]. Another case reported of a patient who developed a systemic AA amyloidosis due to a solitary lung metastasis from RCC, but the primary cancer was completely resected 8 years ahead [10]. Besides those two case reports, all the reported cases of the co-existence of amyloidosis and RCC to date have occurred in localised renal tumours [13].

Untreated, AA amyloidosis is a severe disease with a high morbidity and mortality due to end-stage renal disease [1, 15, 16]. In a review of 374 cases of AA amyloidosis, the mean survival time from diagnosis to death was 133 months. Poor prognostic factors were: elderly, hypoalbuminaemia, baseline end-stage renal failure and elevated SAA during follow-up [1, 16]. Effective treatment of the inflammatory cause, including tumour surgical resection can lead to the stabilisation or improvement in renal function, proteinuria reduction and partial resolution of amyloid deposits. Either partial or complete remission of amyloidosis was achieved in the majority of the cases after removal of the primary tumour [17–21].

The relevance of the current report is based on the presence of high-volume metastatic RCC. The complexity in diagnosing this syndrome in our patient was due to his treatment with Pazopanib during the previous 5 months. A common side-effect of tyrosine-kinase inhibitors is proteinuria, thus the complexity of diagnosis as there was no proteinuria control while receiving it. Due to the severe proteinuria that contraindicated the standard second-line treatment with Nivolumab or Cabozantinib based on data from Checkmate 025 and Meteor clinical trials [22, 23], a palliative cytoreductive nephrectomy was performed to rapidly decrease tumour burden and systemic inflammatory status. Unfortunately, in our patient, debulking surgery did not achieve stabilisation or regression of the systemic AA amyloidosis. Probably, our current scenario predicted a fatal fate as per several organ amyloidosis. Besides, nephrectomy was able to decrease tumour burden but several other locations persisted.

## Conclusion

AA amyloidosis is a serious and rare complication described in malignancies. RCC is the most common solid organ malignancy to cause this serious problem. Rigorous treatment of the underlying cause is necessary to decrease the inflammatory status, main driver of the condition.

Sparse reported cases up to date suggest that nephrectomy can prevent further amyloid deposits and, in some cases, regression might be achieved. Previous reports were in localised renal tumours without metastatic burden. It seems to suggest that not achieving a complete tumour removal on a baseline advanced systemic amyloidosis is a poor prognosis factor.

## List of abbreviations

SAA, Serum amyloid A protein; RCC, Renal cell carcinoma; IL-1, Interleukin 1; IL-6, Interleukin 6; TNF-a, Tumour necrosis factor-a; ANA, Anti-nuclear antibody; ANCA, Anti-neutrophil cytoplasmic antibody; IgA, Immunoglobulin A; IgG, Immunoglobulin G.

## Compliance with ethical standards

### Conflicts of interest

The authors declare that they have no conflict of interest.

### Informed consent

Verbal informed consent was obtained from the patient included in the case report during hospital admission.

## Funding statement

The authors did not receive any funding for this work.
